# Combined Oxygen-Ozone Therapy for Mesh Skin Graft in a Cat with a Hindlimb Extensive Wound

**DOI:** 10.3390/ani13030513

**Published:** 2023-02-01

**Authors:** Nicuşor-Valentin Oros, Călin Repciuc, Ciprian Ober, Mihaela Mihai, Liviu-Ioan Oana

**Affiliations:** 1 Department of Surgery, Faculty of Veterinary Medicine, University of Agricultural Sciences and Veterinary Medicine, 400372 Cluj-Napoca, Romania; 2 Department of Transversal Competencies, University of Agricultural Sciences and Veterinary Medicine of Cluj-Napoca, 400372 Cluj-Napoca, Romania

**Keywords:** wound, cat, hind limb, ozone therapy, mesh skin graft

## Abstract

**Simple Summary:**

Wounds of the extremities in cats accompanied by considerable loss of substance are frequently encountered in current practice and their therapeutic management and choice of closing method is a challenge for the veterinarian while also exerting an influence on the evolution of healing. In this case report, a new complementary technique is proposed, which involved supporting a skin graft using ozone therapy both before and after applying the graft. This approach accelerated the recovery of the patient and no complications were observed.

**Abstract:**

This case report describes a new therapeutic approach for a domestic shorthaired female cat, who has an extensive posttraumatic wound in the right hind limb. After patient stabilization, general anesthesia was started and the wound was cleaned and debrided of devitalized tissues, followed by the application of ozone therapy and bandage. Eight sessions of ozone therapy were performed for 17 days until the application of the skin graft. Three more sessions of ozone therapy were performed every 3 days postoperatively. The bagging method and the perilesional infiltration method were used. The ozone therapy ensured an accelerated recovery of the patient without any complications. According to our knowledge, this is the first case report with the use of ozone therapy to support a free skin graft in a cat. The new therapeutic approach could be used to accelerate healing of the wounds with a significant lack of substance, by supporting pre- and post-operative skin grafts.

## 1. Introduction

The management of skin wounds in the extremities poses a challenge for the veterinarian from a surgical point of view, while from a financial point of view, it can generate significant costs for the owner. The main objective in the therapy of cutaneous wounds in cats, with a significant loss of subdermal tissue structures, is the restoration of their homeostasis. If this is achieved, the healing process will follow an upward trend, toward closing the defect as soon as possible to avoid further complications of an infectious nature [1].

The pathophysiology of wound healing involves three successive phases: inflammatory, proliferative, and epithelialization [2]. The application of a skin graft in wounds with a marked lack of substance replaces the epithelialization phase, favoring the recovery of patients in a much shorter time and with fewer complications [1]. In the veterinary literature, there are numerous therapeutic methods for managing wounds with a significant lack of substance, so that they develop a healthy granulation tissue in the shortest possible time, and then be closed by different plastic surgery techniques [2].

Free-skin grafts can be a good choice when managing wounds in limb extremities since the elasticity of the skin at this level is reduced and other plastic techniques are more difficult to achieve [3]. Reaching a favorable outcome in the completion of skin grafts often requires the use of additional therapy to support the graft [4]. However, the graft may not remain viable and the main factors that negatively influence its viability are the infection at the grafting site and the on-site movement of the skin graft [5]. Ozone is an unstable gas consisting of three oxygen atoms, discovered in the middle of the nineteenth century and originally used as a disinfectant agent for water. Subsequently, its first medical use occurred during the First World War in the treatment of soldiers’ gaseous gangrenes, produced by Clostridium spp. [6]. Positive results of ozone therapy were obtained in human medicine for patients with diabetic ulcers in the limbs [7]. As such, human medical use encourages the extrapolation of this therapy to veterinary patients with acute or chronic pathologies in their limbs.

As such, ozone therapy can be used both systemically and locally. Wounds at the extremities are suitable for topical use in the form of ozone water, ozone oil, or direct exposure of tissues to ozone using a polyethylene bag. Additionally, perilesional infiltrations can be used to stimulate the healing process according to the recommendations of the 2020 Madrid Declaration [8]. Specialized literature reveals solid arguments for ozone activity, particularly in improving circulation at the capillary level and oxygenation of tissues as well as the release of growth factors and autocoids necessary for the healing of tissues [9].

In the inflammatory phase, which involves the presence of neutrophils, macrophages, mast cells, platelets, bacteria, and the toxins produced by them, ozone acts mainly as a bacteriostatic agent, as well as a modulator of inflammation [10]. Furthermore, ozone has demonstrated effective antibacterial activity in rats with induced peritonitis [11]. Ozone intraperitoneal administration in rats reduced the lesions of the intestinal mucosa caused by the phenomenon of ischemia-reperfusion associated with the increase of antioxidant enzymes [12]. Blood circulation and oxygenation of ischemia tissues have also been improved as a result of ozone therapy [13]. Studies also show that ozone therapy used as a complementary method improves wound healing, modulates the immune system, and acts as a topical antibacterial agent [14]. Moreover, scarring is noticeably faster by applying ozone therapy compared to conventional management methods due to the positive impact of medical ozone on angiogenesis, fibroblast activity, and collagen production [15,16,17].

The objective of this study is to assess the therapeutic effect of ozone applied to a grafted wound, pre- and post-operative in the distal hind limb of a domestic shorthair cat. The hypothesis of this paper relies on the antibacterial effect, tissue oxygenation capacity, and growth factor releasing activity of ozone applied by topical and local injectable means, as crucial aspects for quick and complication-free healing.

## 2. Case Description

**Figure 1:**
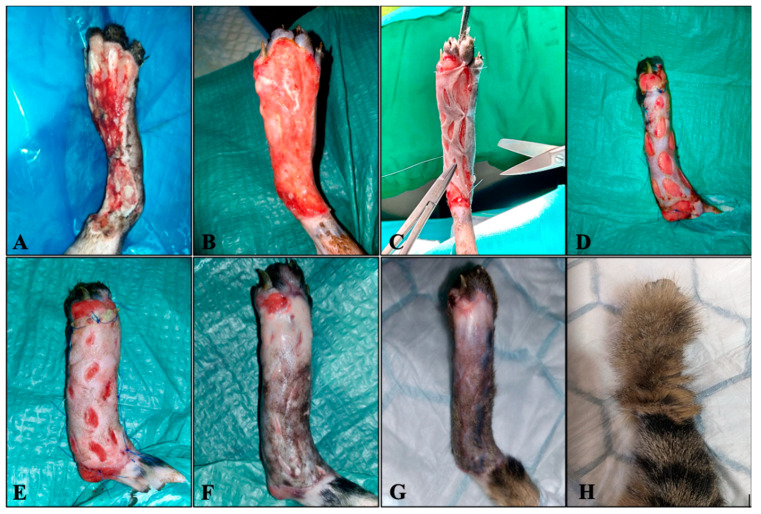
Clinical progression of the wound. (**A**)—Wound appearance at the time of the patient’s appointment at the clinic, areas with necrosis and devitalized tissue, with exposure of the tendons and bones. (**B**)—Wound on day 17 after 8 sessions of ozone therapy with healthy granulation tissue and with the edges of the wound undergoing epithelization. (**C**)—Application of the skin graft. (**D**)—3 days postoperatively. (**E**)—6 days postoperatively. (**F**)—9 days postoperatively. (**G**)—12 days postoperatively. (**H**)—60 days postoperatively (note the longer length and color changes of the new hair).

An adult cat, European short hair breed, intact female, 3.3 kg in weight was evaluated at the Surgery Clinic of the University of Agricultural Sciences and Veterinary Medicine, Cluj-Napoca. The cat required the therapy of a wound with significant substance loss at the level of the right distal hindlimb (Figure 1A). The cat was found on the street and brought to the clinic, with an unknown history. Traumatic origin was assumed, most probably from a car accident. Following the clinical evaluation of the patient, hypothermia of 34.7 °C, a pulse of 180 beats/min, a respiratory rate of 30 breaths/min, and a dehydration score of 15% was revealed. The neurological examination of the patient did not reveal any changes and radiographs revealed no structural changes in the bones and joints. A blood count was performed and regenerative anemia (RBC 3.07 × 1012/L; 5.00 to 10.0012/L, HGB 4.2 g/dL; 8.0 to 15.0 g/dL, HCT 14.99%; 24.00 to 45.00%) and thrombocytopenia PLT (50 × 109/L; 300 to 800 × 109/L) were observed. A rapid FIV-FeLV (FIV Ab/FeLV Ag, VetExpert, Lewes, UK) test was performed, with negative results.

**Figure 2:**
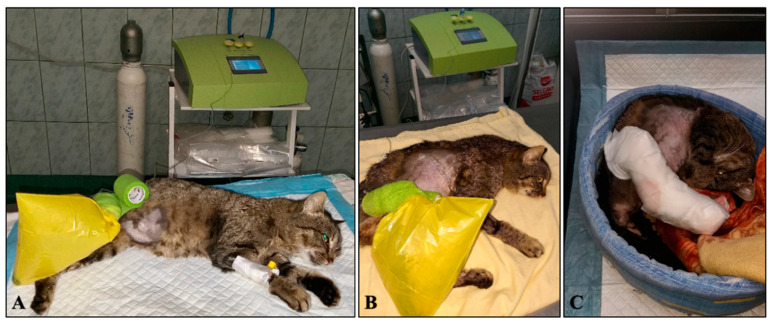
Ozone application. (**A**)—First session. (**B**)—Second session after the skin graft. (**C**)—Dressing applied after skin graft.

Hydroelectrolitic abnormalities were corrected using ringer 10 mL/kg/h (Ringer’s solution, B. Braun, Melsungen AG, Germany) for 4 h. Analgesic medication consisted of buprenorphine 0.02 mg/kg intravenously (IV) (Bupaq 0.3 mg/mL, Richter Pharma, Wels, Austria). After stabilization, the wound was cleaned and debrided under general inhalation anesthesia using isoflurane. Premedication was performed using buprenorphine 0.02 mg/kg IV and ketamine 3 mg/kg IV (Narkamon Bio 10%, Bioveta, Ivanovice na Hane, Czech Republic) followed by propofol 4 mg/kg IV (Propofol-Lipuro 1%, B. Braun Melsungen, Germany). After endotracheal intubation using a 3 mm endotracheal tube (Well Lead Medical Co. Ltd., Guangzhou, China), anesthesia was maintained with isoflurane (Isoflutek 1000 mg/g, Laboratorios Karizoo S.A., Barcelona, Spain) delivered in oxygen (100%), the wound was debrided, and all the necrotic tissue was removed. Copious lavage with 0.9% saline solution was also performed. Samples for culture and sensitivity were obtained. After the completion of the surgical debridement, the total wound surface was determined using a food foil for fingerprinting, respectively the tracing of the lesion with the help of a skin marker and the transcription of this contour on millimeter paper to calculate the surface. The wound covered an area of 51 cm^2^ and about 70% of the diameter of the limb was affected. At the end of the surgery, the wound was treated with ozone (Figure 2A) by polyethylene bag method for 5 min at an ozone concentration of 60 μg/Nml (Microgram/ Normalized milliliter of ozone concentration) and perilesional infiltrations at a concentration of 15 μg/Nml in the amount of 0.3 mL per site of administration using dermal needles of 26 G amounting to a total of 2.1 mL. The wound was covered with a wet-to-dry bandage.

The culture and sensitivity samples revealed Streptococcus spp. alpha-hemolytic, Staphylococcus spp., Bacillus spp., sensitive to marbofloxacin. The cat received marbofloxacin 3 mg/kg orally (PO) (Efex 10 mg, Ceva Santé Animale, Libourne, France) for 5 days, and robenacoxib 1.8 mg/kg PO (Onsior 6 mg, Elanco GmbH, Bad Homburg vor der Höhe, Germany) for 5 days. Ozone therapy was performed pre-operatively for 17 days (Table 1) until an optimal granulation tissue was developed to support the skin graft, respectively the presence of epithelization at the edges of the wound. This indicated that the wound was free of bacterial infection and healthy granulation tissue covering metatarsals and tendinous structures was present.

On the 17th day, the surgical procedure was scheduled. Ozone therapy was performed before surgery, for its vasodilation properties (Table 1) and then the skin graft (Figure 1C) at the lateral level of the right thorax was performed under general anesthesia, using methadone 0.4 mg/kg intramuscularly (IM) (Insistor 10 mg/mL, Richter Pharma, Wels, Austria) as premedication and dexmedetomidine 0.02 mg/kg IV (Sedadex 0.5 mg/mL, Le Vet Beheer, Oudewater, Nederland) 15 min after methadone administration. Induction was achieved with propofol IV (Propofol-Lipuro 1%, B. Braun, Melsungen, Germany) to effect and maintenance with isoflurane (Isoflutek 1000 mg/g, Laboratorios Karizoo S.A., Barcelona, Spain). After rigorous antisepsis of both the receptor site and the donor site, the first step involved the debridement of the wound edges and the removal of the newly formed epithelial tissue. A slight debridement of the granulation tissue was also carried out to create better contact between it and the graft. The second step was to harvest the skin graft after a prior measurement of the necessary skin tissue to cover the defect in the limb. The harvested graft was stretched on a sterile roll of vetrap and fixed to it with syringe needles. The third step was the removal of the subdermal tissue with an 11-blade and then parallel incisions were performed that encompassed its entire thickness. The fourth step was the grafting itself, respectively, the suture of the graft to the edges of the wound in separate points with nylon thread 3-0. Finally, ozone therapy was performed once again for its tissue oxygenating and growth factor-releasing properties (Table 1). A dressing, consisting of sterile compresses, hydrophilic wool, and a gauze bandage was applied to the limb (Figure 2C). 

After graft transplantation, ozone therapy was performed every 3 days, using the bagging method (Figure 2B) at a concentration of 10 μg/Nml with an exposure time of 20 min. In total, there were 3 sessions of post-operative ozone therapy and the graft acceptance rate was about 98%. The ozone for this therapy was produced with a medical generator (Medozon Compact; Herrmann Apparatebau GmbH, Elsenfeld, Germany). The cat was hospitalized for 29 days to complete the therapy and find an adopter. Sixty days after skin grafting, the limb exhibited a normal appearance, without functional changes (Figure 1H). 

## 3. Discussion

Currently, there are no clinical trials to demonstrate that ozone therapy is superior compared to other therapies in the management of skin grafts in cats.

However, ozone administered by infiltration to rats with induced wounds intensifies epithelialization and matrix deposition, while it increases cell proliferation during wound healing. In this present case, it can be observed that on day 17, after 7 sessions of ozone therapy (Figure 1B) the wound presents a healthy intense red granulation tissue, which covers the underlying structures. The edges of the wound are contracted, while the formation of new epithelial tissue begins at the periphery.

Histologically, wounds treated by ozone infiltrations had fewer inflammatory cells and a greater number of fusiform cells in the composition of the granulation tissue [18]. Observations showed that ozone stimulates the contraction phase and improves blood flow in a dehiscence wound in a FiV-positive cat [19]. Our schedule included the constant application of ozone in progressively lower concentrations. Based on the ozone’s hormetic action the 60 micrograms/Nml were used for antibacterial purposes and the lower 10–30 micrograms/Nml concentrations were used for their bioregulating effects [20]. It was shown that constant application of ozone prevents superinfection, and also stimulates cell proliferation, the synthesis of fibronectin, collagen type I and III, hyaluronic acid, and chondroitin sulfate [10].

The antibacterial as well as bacteriostatic properties of ozone are widely studied and scientifically expressed, but the exact mechanisms of action are not completely clear. There is an accepted hypothesis about the oxidative properties of free oxygen radicals that result from the reaction of ozone with wound biological fluids against the bacterial membrane. These products are obtained in higher amounts by using higher medical ozone doses and might be responsible for the lysis of the microbial membrane [21,22,23]. There are also accepted ozonated products represented by oils, water, or saline solution which have been shown to have good bacteriostatic effects [24].

Ozone therapy of the wounds in the distal limb was performed also in human diabetic patients. It was demonstrated that treatments increased the expression of vascular endothelial growth factors (VEGF), platelet-derived growth factor (PDGF), and transformed growth factor β (TGF-β) [7].

A retrospective study evaluating distal limb skin grafts in dogs and cats has shown 77% acceptance in cats (17 out of 22) where 15 of the wounds were localized at the tarsal and metatarsal levels. The same study has concluded an average of 32.1 days is needed for the preoperative treatments and 21 days until complete healing by using bandages of paraffin-impregnated tulle grass, silicone-coated polyamide mesh, hydrocellular foam dressing with nonadherent coating polyurethane top films and different systemic antibiotics for 7 to 37 days [25]. In our case, the duration of open wound treatment before grafting was 17 days and 12 days after grafting until complete healing.

Two other studies evaluating negative pressure wound therapy in two cats with wounds of the same size and localization have reported 26 and 31 (15 and 17 before grafting) days respectively from the first evaluation until complete healing, alongside antibiotic administration throughout the entire treatment period [26,27]. Our patient needed 17 days before grafting and a total of 29 days by only using marbofloxacin in the first 5 days after the primary evaluation and ozone therapy until complete healing, showing that ozone acts as a complementary treatment before and after skin grafting. In this particular case, ozone therapy improved healing, compared to other conventional treatments used in classic procedures, and has at least the same effect as negative pressure wound therapy.

The beneficial activity of ozone in the epithelization phase of the skin graft in our case could be due to the ozone’s ability to release nitric oxide and produce prostacyclin, [28,29]. These have positive effects on the vascular endothelium, respectively on the skin graft by means of a better plasma intake. The viability of the skin graft is dependent on this in the first phase, and then it connects to the underlying tissues through a fibrin network [30,31]. The exposure of red blood cells to different concentrations of ozone leads to an increase in the concentration of tissue oxygen by 2,3-diphosphoglycerate stimulation, as a result of improving the rate of erythrocyte glycolysis [32]. Intrarectal administration at a concentration of 20 μg/mL in rats with induced arterial spasm has beneficial effects on the structure of the blood vessel, partially preventing damage to the vascular endothelium and increasing the diameter of the artery [33]. It is to be noted that cats, compared to dogs, form granulation tissue more slowly and in a smaller amount, which is why it is ideal to use therapies to support this process [34].

Ozone is also known for its high oxidative effects due to hydrogen peroxide production in contact with biological fluids. However, in medical concentrations, it induces a mild transitory oxidative stress, so the hydrogen peroxide has a very short half time which is enough for the stimulation of the antioxidant system of the organism without overwhelming it. These aspects have been shown in studies performed on human blood ozonation with concentrations between 20 and 80 mcg/Nml [10].

The two therapy techniques used for our patient can be achieved at very low costs, as the only important investment is the generator itself. Overall, the therapy sessions involve minor expenses consisting of needles, syringes, polypropylene bags, infusion extensions, and a source of pure medical oxygen, materials that are usually available in every clinic.

According to our knowledge, this is the first report where ozone is used as a complementary therapy to support a skin graft in cats. The beneficial effect of ozone therapy might improve strategies to accelerate the healing of skin grafts in cats.

This is a case report, thus general conclusions are not possible. Future studies should focus on the establishment of therapeutic doses and the moment of application during the healing process. Moreover, in vitro evaluation of the biological effects of ozone therapy as a complementary and regenerative medical procedure effective in the treatment of wounds with a high lack of substance both before and after the application of the skin graft is mandatory. Prospective studies are necessary to validate our results.

## 4. Conclusions

The results of our study support the hypothesis that ozone therapy leads to an accelerated healing process of grafted wounds and is comparable to other conventional treatments used in classic procedures including negative pressure wound therapy.

The favorable and accelerated recovery of our case encourages the use of ozone therapy as a protocol to support skin grafts in cats.

## Figures and Tables

**Table 1:** Ozone therapy schedule.

Day	Session	Method	Ozone Concentration-Time (Minutes)	Observations
0	1	bagging	60 μg/Nml-5	atonic wound, infected with devitalized edges and necrotic tissue
		subcutaneous infiltration	15 μg/Nml	
1	2	bagging	60 μg/Nml-5	exudative wound
		subcutaneous infiltration	15 μg/Nml	
2	3	bagging	60 μg/Nml-5	exudative wound with delimited edges
		subcutaneous infiltration	15 μg/Nml	
5	4	bagging	30 μg/Nml-10	exudative wound with adherent edges to the underlying tissue, without clinical signs of infection
8	5	bagging	20 μg/Nml-10	exudate in small quantities, with fine-grained buds
11	6	bagging	10 μg/Nml-20	homogeneous grain tissue of pale pink color
14	7	bagging	10 μg/Nml-20	uniform granulation tissue with the beginning of epithelialization at the periphery
17	8	bagging	10 μg/Nml-20	application of ozone before surgery
17	9	bagging	10 μg/Nml-20	immediate post-grafting ozone application
20	10	bagging	10 μg/Nml-20	graft of pink skin without pathological exudate
23	11	bagging	10 μg/Nml-20	graft of skin adhering to the underlying tissue and the beginning of epithelization is visible on the periphery of the graft incisions

## Data Availability

Not applicable.
